# Nicotinamide, NAD(P)(H), and Methyl-Group Homeostasis Evolved and Became a Determinant of Ageing Diseases: Hypotheses and Lessons from Pellagra

**DOI:** 10.1155/2012/302875

**Published:** 2012-03-21

**Authors:** Adrian C. Williams, Lisa J. Hill, David B. Ramsden

**Affiliations:** ^1^Neuropharmacology and Neurobiology, School of Clinical and Experimental Medicine, University of Birmingham, Edgbaston, Birmingham B15 2TT, UK; ^2^Institute for Cognitive and Evolutionary Anthropology, University of Oxford, 64 Banbury Road, Oxford OX2 6PN, UK

## Abstract

Compartmentalized redox faults are common to ageing diseases. Dietary constituents are catabolized to NAD(H) donating electrons producing proton-based bioenergy in coevolved, cross-species and cross-organ networks. Nicotinamide and NAD deficiency from poor diet or high expenditure causes pellagra, an ageing and dementing disorder with lost robustness to infection and stress. Nicotinamide and stress induce Nicotinamide-N-methyltransferase (NNMT) improving choline retention but consume methyl groups. High NNMT activity is linked to Parkinson's, cancers, and diseases of affluence. Optimising nicotinamide and choline/methyl group availability is important for brain development and increased during our evolution raising metabolic and methylome ceilings through dietary/metabolic symbiotic means but strict energy constraints remain and life-history tradeoffs are the rule. An optimal energy, NAD and methyl group supply, avoiding hypo and hyper-vitaminoses nicotinamide and choline, is important to healthy ageing and avoids utilising double-edged symbionts or uncontrolled autophagy or reversions to fermentation reactions in inflammatory and cancerous tissue that all redistribute NAD(P)(H), but incur high allostatic costs.

## 1. Introduction

“Progress engenders Leisure whereby Energy is directed toward advancement of the Mind in all parts of Society that in turn receives new Energy.” Turgot, 1750.

“In my theory there is no absolute tendency to Progress, except from favourable Circumstances” Darwin, 1838.

Mental and neurological disorders are poorly explained yet constitute a good proportion of the global burden of disease, now defined as the inability to adapt homeostatically in the face of social, physical, and emotional challenges [1–3]. Robust brain development in the first place must help and is defined by environmental factors and cell types that evolve in a series of networks to ensure the efficient flow of energy and information [4–6]. Energy supplies from food are a prerequisite for producing the necessary variety of cells and simultaneously act as a major selection force influencing their survival; yet despite its importance to brain evolution and development inequalities of diet, particularly over meat and calorific intake, remain a global issue, as do stress responses such as overeating [7–11]. Bidirectional circuits, from the energy supply to terminal fields, are selected from early exuberant developmental (but evolutionarily constrained) fields by environmental exposures but must have weak links as strong safety factors against every eventuality whether environmental, genetic or stochastic would be too expensive in energy terms. At risk circuits may include cholinergic, serotoninergic, and dopaminergic systems that have not scaled up as fast as the overall three-fold expansion of the brain in the human primate [12–20].

Redox energetic faults and aberrant mitochondrial dynamics with consequent oxidative stress, and loss of calcium, glutamate, proteosome, and inflammasome homeostasis are important proximate mechanisms of acute and chronic diseases and the physiological declines that are linked to ageing [21–31]. Some of these disease circuits may have particularly high energy requirements, for instance, those affecting higher intellectual and complex physical exploratory or reward functions using cholinergic or dopaminergic neurons: these for instance have mechanisms of autonomous pace-making with a high metabolic energy cost in terms of ATP to maintain tight control of intracellular calcium and complex redox needs involving cofactors, metals, and melanin [21, 32]. Confirmation of this fundamental and compartmentalized failure of the energy supply, often with impaired autophagy and mitophagy, comes from study of acute brain injury from hypoxia or hypoglycaemia or direct trauma, mitochondrial mutations linked with chronic diseases of ageing such as Alzheimer's (AD), Parkinson's (PD) and Huntington's Disease, and, Complex 1 toxins (such as 1-methyl-4-phenyl-1,2,3,6-tetrahydropyridine (MPTP)) or rotenone and physical tests of endurance such as polar and high altitude expeditions [33–40]. The root cause of these diseases may be exposed energy and informatic links and responses with inadequate redox and mitochondrial potentials affecting proton based power and available ATP. Levels of cellular ATP affect all processes from muscle contraction to repolarization of neuronal membranes, to synthesis of cellular building blocks, to protection against microbes and toxins, and maintenance of proteins, RNA, DNA, and chromatin [41–44].

Now is an appropriate time to look at the effects of energy and the interlinked nicotinamide and methyl-group under and oversupply and their relationships to information transfer and work outputs including those done to improve personal niche environments and evolutionary processes [45, 46]. This survey includes a historical episode when the nicotinamide, and tryptophan and hence NAD(P) supply (and potentially over 400 dependent reactions that influence every area of metabolism) failed as did the supply of methyl-groups. The forgotten case of the complex socioeconomic disease, Pellagra, may be a lesson about losing energy and redox “logic” at several hierarchical levels, and we intend to show that there may be current scope to adjust the dose of the macro- and micronutrients involved, across and within populations and according to individual need, aiming to improve robustness and resilience to a range of degenerative and proliferative diseases [47–49].

**Background (1): Ageing Diseases, Evolution, and NADH**

**Figure 1:**
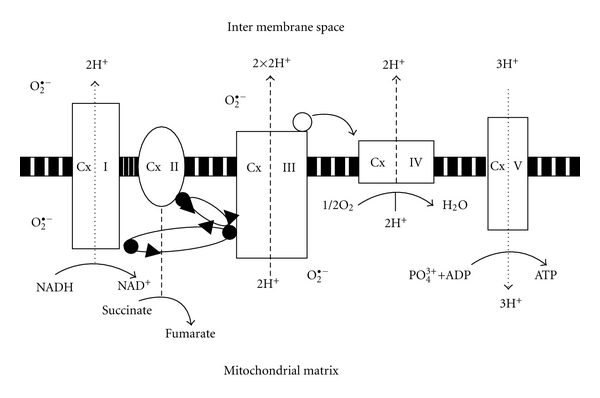
NAD(H)—the electron donor to complex I translocating protons across the membrane by a “steam engine” like mechanism producing ATP: the most important function of mitochondria alongside proton leaks for heat and energy dissipation and signals for autophagy and apoptosis. Many mutations that affect mitochondrial complex 1, or, microtubular function and kinases and autophagy/mitophagy and radical production contribute to rare forms of ageing diseases such as PD in a vicious cycle subverting normal quality controls that affect proteosomal homeostasis. In the case of Parkinsons', increasing NADH levels may drive excessive dopamine synthesis to toxic levels by enhancing the recycling of the cofactor for tyrosine hydroxylase.

Current theories on ageing, lifetime behaviour patterns, reproductive cycles, intergenerational transfer of resource and stress responses invoke energy sources, sensors, regulators and tradeoffs: as do wider theories on the astonishingly rapid metabolic evolution of big brains aided by the ecological, social, and cultural strategies used by man that affect both access to energy useful information and increase in net energy input and allocation of energy resources [50–58]. Thermodynamically open and phylogenomic models support the idea that NADH and nicotinamide adenine dinucleotide phosphate (reduced) (NADPH) have had a central role and are the common currency in linking circadian environments to mitochondrial metabolism and vice versa [59–66] (Figure 1). Sharing hydrogen and its carrier around equitably as the source of electrons is complex relative to the supply of the electron acceptor oxygen that is free, but when successful enables efficient energy flows to be achieved: there is some danger from free radical damage from oxygen metabolism, though a prime importance of this for ageing is now in some dispute with the spotlight now on NAD and nicotinamide [67]. Efficient energy conservation in turn allowed the evolution of complex ecosystems and the expansion of genetic and species diversity, including the development of socially interacting brains working vertically down, up, and across generations often through NAD-dependent neuroendocrine and neurotransmitter mechanisms [68–76].

**Figure 2:**
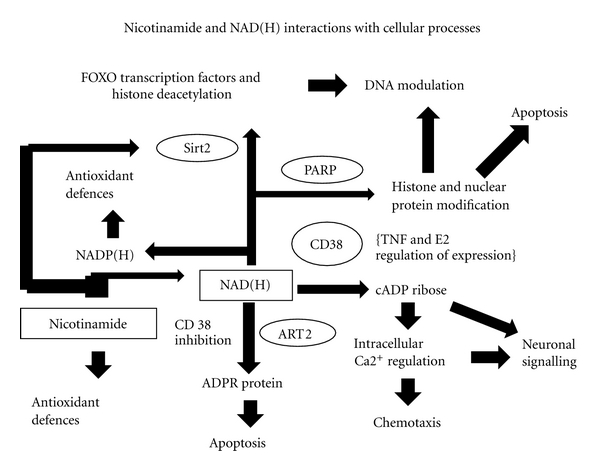
Direct redox regulation apart NAD-consuming pathways influences a prodigious number of pathways involved in internal metabolism and connections with the external world. Survival at the cellular level and of viable interactions with each other and diet and symbionts means that NAD and Nicotinamide are at the hub of the survival of superorganisms such as ourselves. Key: FoxO—forkhead transcription factors; Sirt2 and PARP in text; CD38 cluster differentiation 38; TNF—tumor necrosis factor; E2 Prostaglandin E2; cADP—cyclic adenosine di-phosphate; ART2—T-cell ADPribosyltransferase; ADPR protein—adenosine di-phosphate ribose protein.

Recent reviews on nicotinamide and NAD metabolism, evolution and function concentrate on its pivotal role in gene silencing, development and ageing, through recently appreciated NAD consumers, such as the sirtuins (SIRTs) [77–85], that evolved to work in large part as nutrient-sensing regulators, and poly (ADP ribose) polymerases (PARPs) [86, 87] and on the role of NAD(H) in Wallerian degeneration and neuronal death after a variety of insults [88, 89] (Figure 2). These reviews do not do justice to the importance of nutritional and symbiotic interfaces or the developmental dynamics of inducing NNMT, which partly regulates NAD(H) via controlling and detoxifying the supply of nicotinamide (vitamin B3), at the cost of losing S-adenosyl-methionine (SAM), the only metabolic methyl donor, or the related role of evolutionary history as the ultimate basis for understanding the proximate pathophysiology of a range of modern diseases [90–96].

**Background (2): Symbiotic Energy Environments**

Flow of energy largely determines the structure of ecosystems via food webs and taste sensors with appetite and quality controls, that avoid toxins but allows self-medication, and involve ATP sensitive autophagy in hypothalamic neurones [97–99]. Diet with concurrent domestication of microbes as gut symbionts (or yeasts to ferment foodstuffs) that break down proteins or indigestible starches or manufacture vitamins, including nicotinamide, has evolved to supply and translocate energy and information via circadian pulses of NAD(H): particular symbionts, for example, buffer particular diets such as those low in meat and dairy consumption or high in plant based carbohydrates [100, 101]. Disturbance of these symbiotic relationships, such as by the over-use of antibiotics, is receiving considerable attention in several conditions that are increasing in incidence currently [102]. Swopping production of vitamins and some catabolic enzymes from personal metabolism to diet or symbionts appears to help find hydrogen-based energy [103]. Diets (that both depend on and have shaped cultural niche constructions, such as farming selected crops, domestication of animals for milk and meat, and cooking) and the entangled symbionts which form the microbiome in the gut are essential synergies for the evolution of human life and energy harvesting directly through NADH/ATP production [104, 105]. Diet and symbionts compensate for each other by acting as coupled recycling systems at many metabolic, immunologic, and behavioural levels, and both are implicated when energy balances go wrong as in the case of obesity, and when both are insufficient, gut cells may even break down their own components to obtain energy [106–111].

Failure of the supply of NAD(H) as a consequence of malnutrition or starvation triggers other attempts to compensate by autocarnivory to avoid apoptosis or necrotic cell death, particularly important for neurones and other cells that are rarely renewed after birth [112, 113]. Immune/inflammasome modulation with such malnutrition also occurs and allows the organism to tolerate microorganisms which are potentially deleterious but are also capable of supplying NAD(H) or its precursors [114]. Reversion to fermentation occurs in inflamed or cancerous tissue, favouring net NADH export (the Warburg phenomenon) and interacts with autophagic mechanisms with recruitment of somatic oncogene mutations and methyl-group sensitive epimutations to achieve this metabolic milieu [115–119]. All the above may be homeostatic short-term attempts to correct a poor local energy/redox environment whether by producing, redistributing, or wasting energy to heat. As another redistributive example, ectopic fat stores, which include uncoupling proton-wasting inflammatory sites with loss of mitochondrial potential, are part of the metabolic syndrome to which populations that have recently undergone a nutrition transition are especially prone [120–123].

## 2. Pellagra

An NAD(H) deficiency state that illustrates the complexity of these coevolved energy networks was observed during the largely forgotten epidemics of pellagra caused by economically determined suboptimal foraging of nicotinamide and tryptophan (tryptophan is converted to NAD and serotonin) and dietary sources of methyl-groups. Such a dietary deficiency may still be important in sections of modern populations and intracellular and microenvironmental deficiency states can also occur from high NAD expenditure with NAD(H) ratios also affected by any disorder that causes hypoxia or hypoglycemia [124, 125].

### 2.1. Clinical Features of Pellagra

Scientific detectives investigating ageing diseases and human evolution should revisit a fork in the intellectual road that happened exactly a century ago for real-life ecological rather than solely laboratory-based clues around the NADH-NAD consumer axis (that are often controversial due to confounding factors in experimental design that try too hard to separate physiological homeostasis and stress responses from ageing or are too genecentric given that most genes in some ecological context or other have survival value) [126–141]. Then the last major epidemic in man occurred (pellagra also occurs in several other carnivorous species such as dogs) with a systems failure of NAD(H) and therefore thermodynamic, NAD-Hub, and methylome homeostasis at all hierarchical levels. The epidemic started and ended socioculturally. Hypovitaminosis B3 caused a premature ageing condition with loss of robustness to chemical, physical, and emotional stress, leading to parkinsonism, dementia, metabolic disease, and cancer. It was conquered by dietary supplementation. Famously, pellagra caused dementia, a characteristic photosensitive dermatitis (Casal's necklace), diarrhea, and death. The cause was real world economics creating NAD(H) gradients across poor populations, and across sexes (as it was twice as common in women) in the cotton- and corn-dependent states of the southeastern USA that reached epidemic proportions. Too much corn and molasses and too little meat led to a diet deficient in nicotinamide and probably other vitamins such as riboflavin and choline but often adequate or even high in calories. Currently a similar situation exists in parts of Africa. In the USA, familial aggregations were common, suggesting genetic predisposing factors and widespread epigenetic reprogramming. A diet with too much corn and too little meat caused deficiencies of nicotinamide and the essential amino acid tryptophan, though deficiencies in other vitamins, such as choline, and other methyl donors contributed to the malaise. Mental symptoms were wider than dementia, in that depression, fatigue, psychomotor retardation, mania, obsessions, and a whole range of psychoses with auditory and visual hallucinations were well described, along with personality change and sociopathic and drug and alcohol addictive behaviours. Panic disorders were seen as was a general inability to deal with physical or mental stress. Poor brain development such as hydrocephalus or cerebral palsy was also common. Acute delirium or even coma occurred, with some patients having myoclonus and other extrapyramidal signs reminiscent of the spongiform encephalopathies. The dementias of pellagra included features akin to Lewy body, Alzheimer's, frontotemporal, vascular, and prion diseases. Parkinsonism was also common and a festinant gait was first described in pellagrins. Tremors of various descriptions, including asymmetric rest tremors, were noted and some patients had typical paralysis agitans. Pellagrins had a characteristic expressionless facies, so some signs of parkinsonism were present in most cases. Many features of pellagra closely resemble the nonmotor aspects of PD.

The neurological manifestation did not stop there because other degenerative conditions, such as an amyotrophic lateral sclerosis-like picture, were described, with fasciculation of the tongue and upper and lower motor neuron signs. Cerebellar syndromes occurred and vertigo was frequent. Headaches, sensory and pain syndromes, epilepsy, and involuntary movements were noted as well as sleep disturbances. Cord lesions were also seen, as was optic atrophy, so there were multiple sclerosis (MS), like variants.

Non-neurological effects included the diagnostic photo-sensitive and ageing dermatitis along with diarrhea from multiple gut infections. Other infections including tuberculosis (TB) were common, suggesting a widespread effect on the immune system. There were also profound metabolic effects on the endocrine and thermoregulatory systems. For instance, endocrine dysfunction in thyroid and cortisol and reproductive pathways were all clearly described. Cancer rates were increased but masked by a high premature death rate.

Pellagra without dermatitis was well accepted and a frequently overlooked diagnosis. Young men from affected states had inordinate difficulty in passing the intellectual tests that involved reading, writing and numeracy skills and the physical fitness tests required by the military, suggesting that subclinical disease was rife. Indeed poor diet and health have been implicated in the Southern states both having a tradition of violence (particularly toward the stealing of nicotinamide-rich livestock) and losing the American Civil War [142].

The classic pathological sign was chromatolysis of the high energy requiring pyramidal cells in the cortical gray matter but Purkinje cells also degenerated and there was a widespread cell loss in basal and sympathetic ganglia. Demyelination was commonly seen in the spinal cord, particularly in the posterior and lateral columns. Much was made of pigmentary change, glial overgrowth, vascular change, amyloid deposition, cytoskeletal and mitochondrial abnormalities and signs of inflammation and atrophy in all organs. A systems failure with the degenerative pathology of precocious senility was proposed by several pathologists in the late 19th century, and clinicians commented on patients as “wrecks of humanity”.

Pellagra is a wide systems failure in that an ecologic dietary stress triggers developmental and degenerative effects affecting behaviour and a vicious cycle which amplified the original economic stress. In addition, healthy members of society were phobic about pellagrins and showed them little if any empathy (unlike most other disabilities) and preferred explanations, such as a poor gene pool or that they were infectious, rather than blaming poverty. This picture, we suggest, may represent some aspects of human evolution gone wrong given that not only individuals but also their social relationships were all “wrecked”.

### 2.2. Is Subclinical Pellagra Relevant to Contemporary Disease?

Many poor individuals and groups, even in rich communities, have low amounts of vitamins in their diet. This assumes that the recommended daily allowance (RDA) for vitamins is correct for good health, as opposed to simply avoiding deficiency states. Consequently, subpellagrous nicotinamide deficiency may have lifetime roles in a range of behavioural traits, neuropsychiatric diseases, and dementias. Dietary choline deficiency exacerbates nicotinamide deficiency because metabolism of the two agents is linked; N-methylnicotinamide blocks choline export [143, 144]. Currently, trials of nicotinamide are underway in AD, stroke and several other neurological conditions based on the following [144–182].

1. The brain uses a lot of energy proportionate to its weight and glucose and oxygen status and NADH modulates memory retrieval.
2. In prospective studies, diets of AD patients were found to be deficient in nicotinamide premorbidly.
3. Nicotinamide reduces the accumulation of amyloid by an effect on secretases and affects the toxic effects of amyloid on astrocytes by inhibiting PARP and the NAD synthase nicotinamide mononucleotide adenylyl transferase (NMNAT) that has protein-protein chaperone activity and also affects Tau-induced neurodegeneration by promoting clearance of hyperphosphorylated Tau oligomers.
4. In transgenic mice, nicotinamide restores cognitive deficits with effects on both dendrites and axons and reduces a phosphospecies of tau which is linked to microtubule depolymerisation, an effect also seen following the inhibition of SIRT1, and increases acetylated alpha-tubulin which is linked to microtubule and tau stability.
5. In models of AD, overexpression of SIRT1 or PARPs reduces memory loss and neurodegeneration.
6. Loss of SIRT1 is associated with the accumulation of amyloid-beta and hyperphosphorylated tau in the cortex of AD brains (thought to be compensatory changes to restore glucose/ketone metabolism).
7. Recovery of learning and memory has been associated with chromatin remodeling that happens with agents that affect SIRT and methyl metabolism and epigenetic mechanisms seem to be important in AD.
8. Energy paths involving cAMP and insulin-like growth factors are involved in memory consolidation and enhancement.
9. Dementia is being linked with both being under and over weight stress and exercise levels and the metabolic syndrome.
10. Nicotinamide reduces ischaemic and traumatic brain damage directly and via enhancement of the synthesis of NAD: both are common additional factors in many forms of dementia.
11. Redox active methylene blue affects dementia pathology and its clinical manifestations.
12. SIRT I regulates Notch signaling that is involved with mechanisms of action of mutations in the PSEN1 gene and the proliferation of neural progenitors in the subventricular zone important in the development and evolution of large cortical mantles and essential for cell specification and tissue patterning (and is involved in human cancer).
13. Nicotinamide and SIRTs are involved with promoting DNA repair particularly in oxidative stress circumstances.
14. Poor diet and chronic diarrhea affecting brain function may also interact with tradeoffs at genetic and antagonistic pleiotropic levels. Two copies of the e4 allele of apolipoprotein E lower the incidence of childhood diarrhea and poor cognitive development but increase the risk of AD later and appear to be within the reach of natural selection as do other genes affecting human longevity.
15. Lysosomal proteolysis and autophagy require presenilin 1 and are disrupted by Alzheimer mutations and PD-related mutations.

The neurology of HIV/AIDS, which includes premature ageing, dementia, and parkinsonism has clinical, pathological and biochemical similarities with pellagra [183]: many at-risk groups are nicotinamide deficient given that monophagic corn-based diets are now common in Africa. Both AIDS sufferers and pellagrins get many parasitic infections which may be dangerous homeostatic responses that boost NAD(H) in the host when dietary sources of energy are poor. Tuberculosis, for instance, excretes nicotinamide, is inhibited by nicotinamide and related compounds such as Isoniazid and treatment can precipitate pellagra and many gut infections/parasites break down otherwise indigestible cellulose, ultimately producing NAD(H) [184]. The HIV disease marker CD38 is a NADase that regulates intracellular NAD(H), implying disturbed nicotinamide-NAD(H) metabolism [185]. SIRTs regulate the HIV transactivator Tat, promoting viral transcription and, through the NF-kappaB complex, induce T-cell activation particularly in dendritic cells and there are other links between NAD and the immune system [186, 187]. In view of the inhibitory effect of nicotinamide on SIRT activation and other actions, nicotinamide ought to be protective in AIDS and other chronic infections such as TB (that were particularly common in pellagrins) as an important missing host factor that affects virulence acting much like a vaccine [188]. Interaction between symbionts, such as the tuberculosis bacillus, happens by modulation of immune responses via tryptophan-NAD and mTOR pathways in dendritic and Th17 cells that have been under significant evolutionary pressures and span immune and energy pathways, even using ATP as a danger signal [189–193]. The tryptophan oxidation pathway is particularly interesting in the current context as it is both the de novo synthetic pathway for NAD (nicotinamide is like choline a semivitamin) and the tolerogenic pathway both for microbes and cancer and contains some potential neurotoxins and is disturbed in many degenerative diseases: enough NAD from vitamin B3 sources means less toleration of symbionts and at the extreme immune intolerance and allergic disorders [194, 195].

## 3. Hypervitaminosis B3 and Choline

Macronutrients and micronutrients, which include nicotinamide, choline, and other methyl sources, may all obey Bertrand's rule in that they have an inverted “U” shaped dose-response curve with toxicity at either extreme, as was suggested by Waaler regarding increased mortality at either extreme of weight/height ratio curves [196]. Affluent populations are exposed to diets with a historically high calorific content from refined sugars and fats relative to energy expenditure. In these populations, energy expenditure is relatively low because of limited exercise and the fact that body heat is maintained through living in artificial environments supported by the use of fossil fuels. Damage is often assumed to occur from the effects of an obesogenic environment rather than the more direct effect of disturbed NAD^+^ : NADH ratios on a variety of dependent insulin, cortisol and other pathways (also affected by other bad habits such as smoking and alcohol and stress) with obesity being an attempt to sequester energy and actively metabolise nicotinamide (more in keeping with its biological buffering role against starvation, even if it comes at a price) [197–199]. The relationship of the dose of the hydrogen source (caloric intake) with the dose of the suppliers of the carrier (NAD), derived ultimately from nicotinamide and tryptophan, that must be important for high quality biologically useful energy, has never been investigated in detail. Recently the possibility of both too little and too much choline in diet, modified by gut flora and genetic factors, has been raised in the context of vascular disease [200–202].

Extra choline and other methyl-group sources in diet from vegetables, meat, milk, and eggs during our evolution just like other key molecules such as nicotinamide would have enabled the development of cholinergic, glutaminergic, and monoaminergic systems that have actively evolved in primates and through, for instance, the NADH-dependent recycling of an essential cofactor, tetrahydrobiopterin, remain dependent on a good NADH supply for adequate learning and social and reward behaviour involved in sustenance and in desiring advance information, often mediated by dopamine [203–207]. Oxytocin/Vasopressin pathways are also important for the social interactions that have been so important to our evolution as are social substances such as alcohol which are also NAD-dependent, but all these systems may leave us prone to overindulgence as circumstances have improved [208–211].

## 4. An Evolutionary Perspective: Nutritional and Cognitive Transitions

An evolutionary perspective is warranted, because ageing of the cerebral cortex and basal ganglia is more prominent in humans than other primates, who also do not get AD or PD naturally (even though toxins such as MPTP produce a PD-like phenotype in the laboratory) and as living to old ages appears to have arrived late in our evolution and the ageing process may even finally stop [212–215]. High exposure to nicotinamide and choline and other dietary changes that boosted the overall supply of NAD(P)H may have been important ingredients of the change in diet of primates from a grass eating or herbivorous and fruit eating one toward one containing more starch from tubers and vegetables on to meat/marrow/brain eating. This triggered the 3-fold growth over 3 million years of the modern human brain and a high degree of control over the energy environment [19, 210–220].

This changed diet towards high quality energy (not just extra calories from “junk” food, because it combines high calorific content with key vitamins such as nicotinamide and several methyl donors (choline/folate/B12) and essential amino acids such as tryptophan and phenylalanine and lipid components such as Omega-3 fatty acids from fish [221, 222]). This change first happened in NAD(H) source rich patches of the less arid parts of Africa with repeated genetic and informatic bottle-necking as our species followed energy trails out of Africa and was facilitated by the original invention of tools for butchery and later by hunting in groups and by the sharing and storing of meat [221]. Significant brain power is necessary for these group behaviours where individuals specialize and complement each other and read and anticipate minds of other hunters and prey and animal assistants with special sensory skills, and, learn to avoid toxic fauna and find natural medication now in a more seasonal and unpredictable environment. This brain power enables future-orientated thinking and delayed reward discounting, so that habitats or relationships were enhanced and not destroyed for short-term gain [223–227].

Preadaptations such as endothermy and earlier modifications of aerobic mitochondrial function enabling proton leaks and an ample blood flow to the brain to keep it cool for diurnal and persistent hunting may have helped [228]. Hominids, from Homo Erectus on, clearly went for acquiring multiple high energy sources. This enabled the resultant evolutionary advances via a positive feedback loop in which increasing energy sources that were easier to chew allowed the development of larger brains (but small teeth, masseters and guts), which in turn increased needs, which led to the search for more and greater energy sources as niche construction and included the use of exosomatic energy. However, our brain size may have peaked and it is smaller than the more carnivorous Neanderthals. In several species, domestication can lead to striking reductions in both cortical and striatal volumes surprisingly quickly, showing that this is a fluid process [229]. The invention of farming of grain and particularly meat and milk suppliers, followed by corresponding nutrigenomic innovations, that facilitated adaptations to new NADH-rich environmental opportunity, affecting lactose (milk is a valuable source of calories, pathogen-free fluid, and nicotinamide) and alcohol tolerance, amylase activity and nicotinamide methylation, happened on multiple occasions and spread fast [227–230]. This suggests very strong selection pressures working through both cultural and genetic inheritance, as does the rapid evolution and diversity of genes involved with mitochondrial function and glycolytic pathways [231]. This pressure seems to be every bit as important as mutational changes in genes directly affecting brain function [232–235].

Bipedal man is thought to have adapted fast, allowing time and energy to be freed up along with sedentism that in itself reduced energy expenditure from personal and child transport and by having a roof over their heads [236]. The resultant leisure led to the advancement of interactive minds and a distributed intelligence through multigenerational and multidirectional social structures with critical mass—important for divisions of labour—and a series of information revolutions that continue to this day (the “grandmother hypothesis” favours informatic and energy transfer but grandchildren may equally facilitate vertical transfers of certain resources such as new language or technological skills and have been an underrated factor in social cohesion and longevity).

Along with the social transmission of food preferences often mediated by acetylcholine release, which reduced the fear of novel foods, these information revolutions improved decisions over the trading of and even inheritance patterns of energy sources and other materials [237, 238]. Intolerance of a restricted diet based on “fall-back” foods may also explain our love of flavour and the difficulty of finding single foods that are particularly protective against diseases rather than well-balanced omnivorous mixtures. The preference for a broad diet and the danger inherent in monophagy are evidenced by the many phenotypes seen with pellagra [239].

All in turn allowed advanced niche construction and the development of an energy-rich environment, which was enhanced by cooking that releases bioavailable nicotinamide unlike some other vitamins that are destroyed by cooking. Combined with other uses of fire, this enabled a more ecologically diverse and productive environment, such as increasing the number of available herbivores, so that less energy was expended on hunting but with greater certainty of success. Early man, through some degree of social independence and a consciousness, could move to brand new pastures or construct pastures new through planning, imagination, and innovation [240, 241].

## 5. More Recent History: The Neolithic and the Columbian Exchange

The history of maize, the crop that triggered pellagra, is a good example of coevolution because it is now dependent upon us for its sexual reproduction and we are very dependent on it as an energy source. The initial cultural spread of maize is one of the best examples of diffusion of innovation, which sometimes went wrong from lack of attention to detail. When it was imported to the southeastern states around 2000BC and later Europe from central Mexico as part of the “Columbian exchange”, it was improperly cooked and not combined with beans and squash or adequate meat. Maize is popular as it is a C4 plant with very efficient photosynthesis particularly when water is at a premium. New breeds underpin a large section of modern agribusiness. Together with other technoinnovations they have raised production from 1 Kg per hour on a Kenyan farm to 1000 Kg per hour on farms in Iowa [242].

Maintaining and enhancing the supply of high quality components, such as meat and milk, during the original agricultural revolution/Neolithic Demographic transition and over recent centuries has not always been easy and has caused tradeoffs between high population growth and health [243–246]. At times, micronutrient shortage such as lack of nicotinamide would have been more of a problem than shortage of calories and may be a mirror image of the much more recent contemporary demographic transition in Western industrialized societies. Population growth may have had a large part to play in the chronic malnutrition that has stunted growth and led populations to be prone to acute infection (which stresses the NADH axis); it has coincided with the coevolution of chronic infections (e.g., TB that excretes nicotinamide) and malaria (that affects NADH status, as do malaria resistance genes such as those causing G-6PD deficiency).

## 6. Affluence

 A doubling of life span has occurred in many economies over the last 200 years, largely from the reduction in what were then common infectious diseases. Better diet has increased resistance to acute infections that compete for NAD resources as do their toxins. In the case of the so-called “infectious chronic unconquerables”, such as malaria and TB, there appears to be an immunological stand-off in which a degree of tolerance may have been traded for an increased supply of an essential nutrient. Many apparently healthy carriers, which were present in the dietary deficient population, literally disappeared as dietary standards improved in Europe and the USA. Thus, with the increasing quality of diet, symbionts become no longer necessary to supplement diet and the immune system can revert from controlling the population size of a mutualist parasite to trying to kill every invader. Recent information on the role of NAD modulating innate immunity suggests complex energy-centred interactions between neuronal and immunological systems. Contextually useful organisms (as seen in cases of pellagra) depend on ecological factors such as diet rather than geography or phylogenetic considerations, and on a good diet with changed prebiotic composition may no longer be advantageous. With the improved western diet, the immunological reaction to the formerly “useful” and actively welcomed biotic antigens concerned may be “confused” and may extend through molecular mimicry and nutrition-related signals to other antigens, thus setting off abnormal inflammatory and autoimmune reactions including to foods and pollens (that contain NAD(P)H oxidases). This is a different explanation to the more restricted hygiene hypothesis where lack of exposure rather than actively shunning “old friends” is postulated to cause immunological hyperreactivity [247–251].

Here we suggest the possibility that a hypervitaminosis B3 state, with both nicotinamide and N-methylnicotinamide having an optimal range, resulting from a western diet overrich in meat and vitamin supplements working alongside calorific excesses may be implicated in other modern disease phenomena. Meat eating is preferred where it is available, though during pregnancy it is a common dietary aversion, suggesting that it can be toxic in early development as does the presence of the detoxification enzyme, NNMT and evidence of acute toxicity with nicotinamide overdose and worries about red meat being a risk factor for some cancers [252–254]. Nicotinamide exposure can be high, in excess of five times the recommended daily amount (15 mg/day) where diet has both cereals and “high energy” drinks supplemented with the vitamin and where there is an abundance of cheap meat and milk. This chronic overload can be envisioned as working through unbalancing NAD(H)-dependent systems, including SIRTs and PARPs or dehydrogenase pathways (for instance in cortisol metabolism), and through the direct effects of nicotinamide or N-methylated metabolites. NAD^+^ is the substrate for SIRTs and nicotinamide is an inhibitor, so dietary levels of NAD(H) precursors could act as both positive and negative influences on such systems. In the case of nicotinamide, dietary inputs are modulated in part by genetic determination of NNMT levels, with 24% of the population being high expressors. Nongenetic factors also modulate expression of the enzyme. These include nicotinamide itself, stress and the demands imposed on NAD(H) availability by growth, tissue repair and exercise. Interestingly, NNMT and other components of NAD(H) pathways are markedly induced in a variety of currently common cancers as well as in the metabolic syndrome, obesity, PD and autism [255–262].

 An animal model of toxicity from excess dietary nicotinamide has been reported. In this model, dopaminergic neurons were damaged and locomotor activity was reduced. In addition, this model responded to L-DOPA [263]. In other models, nicotinamide and novel derivatives such as N-Nicotinoyl dopamine can reduce melanin as happens in PD [264, 265].

One consequence of NNMT induction and other methyl-transferase reactions is a depletion of methyl stores and S-adenosylmethionine (SAM) that is ultimately dependent on an adequate dietary supply from expensive vegetables and meat rather than cheaper cereals such as corn (banned in 19th century France as unfit for human consumption, thus eliminating pellagra at a stroke: a lesson for modern Africa) [266]. Depletion of available methyl groups, whether from a poor diet or one with excessive nicotinamide, may be a common cause of epigenetic phenomena given the marked influence methylation has on gene expression in pluripotent and differentiated cells that maintain cellular identity [265]. The methylome is an important mediator of information and modifies gene transcription and translation including riboswitches affecting RNA metabolism and in toxicology pathways in concert with NAD and ATP and all appears to be involved in a number of diseases seen within the pellagra phenotype such as cancer and motor neurone disease [267–272]. A major role for the methylome is well accepted for many cancers and their related epimutations sometimes working in concert with DNA sequence mutations in tumour suppressor and DNA repair genes and may well be modifiable by diet [273–277]. There may be several mechanisms whereby such metabolic programming can be inherited and affect offspring [278].

## 7. A Deep History of Hydrogen and Hydrogen Symbioses: Origin of Cooperative Species [279–281]

Life without hydrogen is impossible, whether free or bound with NAD(P) as energy or with oxygen as a solvent. The sun burns hydrogen in a fusion reaction and emits light energy. Early life chemo-litho-autotrophs used redox gradients including hydrogen as the electron donor and then the earliest animals developed symbiotic relationships with bacteria that produced hydrogen and the invention of photosynthesis split water to produce hydrogen as NADPH and oxygen (earlier forms split H2S). Hydrogen can escape from the earth and led to the great oxidation events and an oxygen atmosphere that along with important symbiotic acquisitions involving chloroplasts, hydrogenosomes, and mitochondria allowed the evolution of complex plants and the rise of an animal kingdom that eats hydrogen sources or has gut symbionts that produce hydrogen equivalents from otherwise indigestible foods. Too much hydrogen escape from splitting of water by radiation in the upper atmosphere creates “runaway greenhouse” effects that are incompatible with life and may have happened on other planets and could happen here. Early mammalian inventions that would increase hydrogen and nicotinamide sources include placental and milk feeding. Healthy energy ecosystems reduce poverty and underpin healthy interactive economies: and even though modern economies appear to move away from a primary reliance on natural capital and capturing solar energy, we all need to eat well to stay healthy and need to appreciate the real source of our prosperity and the need to avoid food crises worldwide. 

## 8. Longevity and Nutritional Balance

The NAD(H) energy path and NAD consuming hubs are involved in many key cellular processes and in the regulation of food intake and the phenomenon of caloric restriction and hormesis (that which does not kill you, whether diet, toxin, emotional, acute infectious or traumatic stress, makes you stronger) which are implicated in many diseases of ageing [282–302]. These hubs are amenable to therapeutic intervention and may unwittingly have contributed by a better overall diet and energy budgets to recent increases in longevity in affluent countries (Figure 2). Age-related changes in NAD/NADH ratios have been noted and a world where NAD is central postulated [303, 304]. Placebo effects that are so prominent in man also have been linked to energy pathways and are particularly effective when the energy future is perceived as healthy, allowing energy to be invested in healing rather than fire fighting [305]. Endocannaboids that depend upon the organisms energy status may translate food availability into fundamental choices about development which affect lifespan, and some addictions that were common in pellagrins such as Nicotine are also closely linked to energy pathways. Caloric restriction hints at the need for a nutritional stoichiometry to balance energy demand and supply throughout life. It is associated with telomeric stability and increased SIRT 1 activity stabilising PGC-1 and results in increased mitochondrial biogenesis, metabolic function, and the deacetylation and inactivation p53. This balance may be more about NAD^+^  : NADH ratios than calories per se, which may explain the perplexing and contradictory role of NAD-consumer agonists (such as resveratrol) and antagonists in disease models. SIRT1 knockouts show widespread p53 activation and shortened life expectancy [306]. Increased longevity as a result of caloric restriction and related genetic models fits with abundant evidence that nicotinamide is involved in neuroprotection [307–318]. The damaging effects of too low a dose are a matter of historical record. Evidence is emerging on excessive intake of the vitamin [319, 320]. Nicotinamide's actions may be double-edged as although it is efficacious in a wide range of current insults and pathologies too much may be as harmful as too little.

Links between nicotinamide, acetyl and methyl metabolism and epigenetic phenomena are strong [319, 320]. Other recently studied famines such as the Dutch hunger winter of 1944-45 and the Chinese famine of 1958–61 had marked long-term and transgenerational effects on health such as the risk of the metabolic syndrome but also psychiatric and antisocial behaviours working through epigenetic mechanisms: those affected would have been NAD(H)-deficient with an element of pellagra [321, 322]. Optimising choline and other aspects of methyl metabolism in embryonic life improves brain development and strikingly reduces age-related declines, perhaps through reducing epigenetic instability. Epigenomes, which may be marked by dietary, emotional, and intellectual experiences working through neuroendocrine stress pathways that themselves are redox and energy-regulated and implicated with many “modern” diseases, can be assimilated into the germline and are unstable, creating somatic epimutations during mitoses, some of which may be resistant to erasure. Surviving epigenetic imprints may be the missing transgenerational elements, along with nonshared environments (merging nature and nurture), causing both familial clustering and sporadic cases in diseases such as AD and PD. Twin studies show low concordance in AD and PD, thereby demonstrating the importance of environmental dynamics and chance epigenetic drift over lifetimes [323].

## 9. Development to Decline: Managing Metabolic Expectations

**Figure 3:**
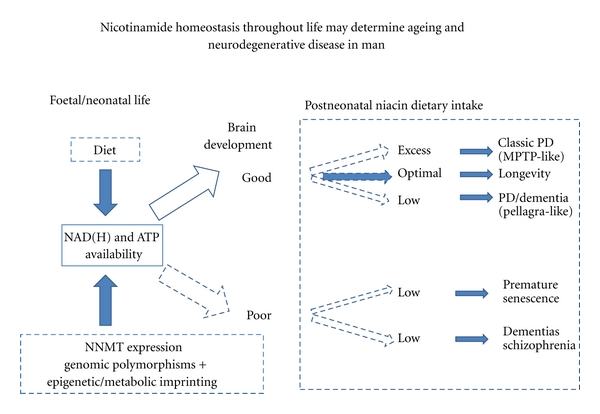
NAD(H) availability is key to good brain evolution and development. An inherent weak link is poor supply or a potential imbalance between induction of NNMT and nicotinamide and choline intake in early and later life with a risk of late degenerations such as Alzheimer's and Parkinson's disease. Overinduction of NNMT by nicotinamide could paradoxically cause intracellular pellagra perhaps explaining why nicotinamide can appear to help short-term even though nicotinamide may be a long-term toxin. In addition, poor diet or induction of NNMT disturbs the methylome and the epigenome creating cancer promoting epimutations.

Within the context of lifetime availability of NAD(H), where in adult life the net free energy or the availability of methyl groups is greater or lesser than that in early life or expected *a priori *by the genome of the individual, an imbalance between (epi)genetic-primed protein inductions and nicotinamide may occur if circumstance overcome tastes, habits, and addictions that attempt to stabilise intake. This imbalance is more nuanced than might be expected and not simply due to the fact that modern diets are mismatched with genetic “thrifty” gene hangovers from the Pleistocene [324]. Our evolutionary history of extreme fluctuations in meat intake from low to high as hunter-gatherers to patchy and still in transition is likely to be more relevant. Nutrigenomic and informatic adaptations and changes in tradeoffs resulting from knowing that the energy future is often more likely to be benign have occurred over the last 100,000 years and during individuals lifetimes. Low levels of nicotinamide and choline in early life adversely affect neonatal brain development and later degeneration [325]. Low NAD(H) levels in the foetal/neonatal period could result in a phenotype which could be reinforced by low levels through life, giving rise to poor brain development and function. High early levels could lead to good brain development (“luxury” phenotype) but a drop in the supply later in life, even to average levels when NNMT, the catabolic enzyme, has been induced, could trigger intracellular pellagra. If the NAD(H) and nicotinamide supply is superabundant throughout, life toxicity might result from excessive N-methyl-nicotinamide, a MPTP lookalike (Figure 3). This is plausible because nicotinamide and redox status have marked morphogenic effects that instruct internally secreted morphogens which regulate all phases of neuronal development and in effect brain evolution [326–328]. The effects can become pathological when unexpected energy circumstances are encountered, as seen with metabolic syndrome, and we propose neurological disease [329].

## 10. Metabolic Fields

Accumulating evidence points to the probability that ageing diseases such as AD, PD, cancers, and metabolic diseases originate in mitochondrial depolarisations and uncouplings with disturbed electron-proton flows as “protonopathies” and have consequent effects on ATP [330–333]. A unifying explanation imagines abnormally spreading metabolic fields, if the challenges of balancing NADH- dependent energy supply and demand and the need for optimal methyl group availability that arises during growth, reproduction, exercise, and tissue repair are not met; most cancers, for instance are demethylated and have changed their energy metabolism profile even in precancerous stages [334]. These challenges could happen at the same stage of life in diverse species, explaining similar incidences of cancer and degeneration despite large variations in numbers of cells/body size and longevity that are hard to explain on a somatic mutational or purely immunological basis which may be secondary or compounding phenomena [335]. Reactions to stabilize metabolism by excreting NAD(P)H (essentially a waste product not a source of energy in cells using anaerobic metabolism) or other reducing equivalents (e.g., lactate), such as autocarnivory/autophagy/apoptosis and the Warburg effect (such that both the oxic and, of course, the anoxic portions of the tumor are both fermenting) could spread like a wave: as do their pathological correlates, whether degenerations, infections, or cancers. NAD metabolizing ectoenzymes and salvage pathways appear to be important in shaping tumor-host interactions with mitochondrial pathways affecting whether or not cancer cells live or are primed for death [255, 336–344]. NNMT activity affects proliferation of cancer cells, affects radiation sensitivity, and alters free radical production; nicotinamide affects genomic instability and cancer cells eat tryptophan [340, 345, 346]. These heavily involved pathways have opened therapeutic possibilities for several tumours including glioblastoma where the importance of mitochondrial dynamics is increasingly recognised [347]. In cancer cells many germline and somatic mutations continue to evolve as cancer spreads, for example, in p53 and relevant receptors, such as those for steroids and growth factor signals. Thus glucose uptake and mitochondrial and isocitrate and phosphoglycerate dehydrogenases and pyruvate kinases are affected, allowing multiple switches from oxidative metabolism to net NAD(P)H production affecting both energetic and redox buffering/antioxidant capacity [262, 348–355]. The paradox here if solely seen from the cancers point of view, rather than viewing this as a pseudo-symbiosis, is that the growing tissue is wasting energy and the argument that it gets ATP faster through glycolysis is weak [356]. Cancers cannot always be eliminated and often recur (though occasionally spontaneously remit) or a second cancer develops, suggesting that the basic problem is not eliminated by simply killing cancer and surrounding normal cells (which may however temporarily improve the local energy environment by releasing ATP and its precursors).

Foetal implants develop PD pathology, illustrating the point that the niche microenvironment is the key [357–363]. Stem cell implants in PD and other conditions survive and connect but form strange lesions and connections suggesting that lack of stem cells is not the real deficit. Rather stem-cell-based therapy from the haematological transplant to those for other organs involves the efflux of NAD and ATP into the extracellular space or autocrine induction of NAD release activating purinergic and calcium pathways affecting growth-supporting and antiapoptotic activities relevant to their therapeutic effects [364]. Parabiotic fusions of young environments rejuvenate cells, even those expressing procancerous somatic mutations or destined for autophagy [365] and changing the ageing milieu, alter neurogenesis and improves cognitive function. Stem cell fates toward both normal and abnormal differentiation or dedifferentiation depend on their microenvironment and stochastic factors, and even cells that are already cancerous can be induced to behave normally [366–369]. Protein folding and the function of amyloid/synuclein/prion proteins and ionic and neurotransmitter gradients are exquisitely sensitive to energy landscapes, as are NAD(H)-consumer linked DNA repairs. Consequently, all these phenomena, when abnormal, may be secondary and late effects of abnormal NAD(H) ratios and methylation status affecting epigenomic phenotypic plasticity.

## 11. Regeneration and Degeneration on Purpose

In complex organisms stem-cell-driven regeneration, which could compensate for long-term damage that is beyond repair, is suppressed particularly in the CNS, whilst dying cells from traumatic injury releasing NADH equivalents may be a potent stimulus for regeneration in other tissues and simpler organisms [370]. This apparent design failure, which is actively pursued by complex organisms, may be rooted in energy allocation models, that is, in tradeoffs that favour building spare capacity during development. Spare capacity when young, which can be coopted as an exaptation for early in life innovative thinking about the environment, may be evolutionarily more advantageous than maintaining, at high cost, regenerative machinery permanently on stand-by that may never get used.

The brain functions within the constraints of an energy (ATP) budget, with high levels of energy used to process information from the environment via action potentials and less energy intensive metabolic processes used to recycle neurotransmitters [371–374]. There is evidence that this budget evolved to permit other tradeoffs allowing neuronal energy costs to remain low while still allowing the organism to gather all required information. In environments where certain sensory information is not needed, evolution has led to the loss of superfluous sensory systems. This responsive and adaptable evolutionary process can also lead to the addition of sensory systems as required by changing environments. Such additions and deletions develop as a result of an advantageous cost/benefit analysis by the evolving organism responding to changing environments and to learn for specific tasks as well as for general intelligence [375].

Poor development from poor early energy circumstances and loss of reserve capacity would be expected to cause late problems, as suspected with AD. Nevertheless, continual regeneration and selection of circuits that are using energy are constantly occurring by altering mitochondrial and SIRT function, and bioelectric free energy and proton fields and fluxes, that influence positional memory and regeneration of even complex structures such as the spinal cord (giving hope that improving the NAD(H) and methyl environment is never too late) [7, 376–378].

## 12. Energy Circuits Can Breakdown

We live in a complex habitat and an energy and informatic subsistence environment largely of our own making that has driven our brain and behaviour patterns to ecological rationality, in order to cope with being far from thermodynamic equilibrium, with strong positive feed-forward decision-making and negative feedback homeostatic mechanisms that drive progress. Less fortunately disease phenotypes from the cancerous to the neuropsychiatric happen when it goes wrong, as happened in the case of pellagra. Pellagra like most famines was not wholly caused by natural disasters but man-made collapses at a series of hierarchical levels from the social to the subcellular. Ever since Homo Erectus, our genus has been addicted to acquiring more and more energy, first as NADH and then harnessing all forms of energy, with a push from coping with poor energy environments and a pull from using excess energy to best advantage through the use of our collective imagination and each other: much current intellectual effort is currently expended on discovering new sources of energy especially those based on hydrogen [379, 380] (Table 1). Many memes/ideas are generated and their survival may often have been dependent upon whether they contain energy-useful ideas, with logic systems working at biological and behavioral levels building on simpler circuits such as the iconic transcriptional regulation of the lac operon in response to changing energy sources. This requires a high quality diet and gut symbionts forming superorganisms with extended metabolisms that are not simply divided by skin or skull from the world but are complementary systems that still have fault lines that can breakdown. Self, as usually defined by the immunologists, is misleading, and more modern views on metagenomes are closer to the mark and, like the psychologist William James, define self as “all a man can call his” and includes his sources of energy and information [381].

Despite consuming a high proportion of the body's energy, brains are efficient (Watson, IBM's latest AI answer machine runs on 100 Kw compared with 100 w for a brain) and lose functions no longer used, suggesting strict constraints on bioenergy allowing the brain to continually evolve along with the changing environment, with considerable potential for human enhancement if the environment improves [382]. Enrichment of the cell's microenvironmental energy ecosystem and a “use it or lose it” strategy leading to energy flow may be key to improvements in the longevity of many cell types and in prompting neural stem cells, “hungry for action”, to divide. Maintaining optimal NADH : NAD^+^ ratios and methylation status through dynamic energy budgets in every compartment is a continual challenge which is awe-inspiring in its complexity. Nevertheless, such considerations are necessary for every single cell if they are to avoid death or cancerous change and may be aided by quantum effects on the distribution of energy [383]. High energy circuits and electrical waves, such as gamma oscillations, are needed for intellectual hippocampal and motor programmes that require strong functional performance of mitochondria and particularly need the pyramidal neurones that take a big hit in pellagra [384, 385]. These circuits are synchronous with circadian NAD(H)-dependent informatic rhythms between individuals and their diets, symbionts and social relationships and beliefs, some mediated by hormones such as oxytocin and neurotransmitters such as dopamine. Sleep gives time for synaptic normalization after dealing with ever-changing environments and unsustainable consumption of energy with saturated abilities to learn as environmentally cued memory and free-will decision making, over and above reflex preactivation of volition, require particularly high energy levels: these are functions hit early in cortical and basal ganglia degenerations some of which compensate by the patients developing a sweet tooth or otherwise modify energy intake or expenditure as part of the symptoms or attempts at treatment [386–396].

These environmentally coupled systems, largely of our own making to keep energy flowing and balancing excitatory and inhibitory circuits, can get decoupled with energy or informatic under or overload (“rewired and running hot”) and may be hit preferentially in PD and AD and other diseases peculiar to Homo sapiens, such as depression and psychoses where nicotinamide aberrations have long been suspected [358, 359]. We urgently need to improve our abilities to measure or image suitable NADH supplies in a series of human habitats on the outsides of our Russian Doll Redox arrangement and NAD(P)H/NAD(P)^+^ ratios in every internal compartment, cell type and organelle as early warning systems to intervene and avoid pathology even getting started.

## 13. Conclusion

Classical pellagra is an archetypal energy and NAD supply-side premature ageing disease, with excessive use of symbionts and degenerative pathology, that has been cured but we have never checked to see whether subpellagrous NADH deficiency has been eliminated. Sub pellagrous NAD deficiency may be a man-made socioeconomic “place” disease that rears its head episodically as a “time” disease triggering age-related infections, degenerations, and cancers [397]. This type of socioeconomic illness does not naturally trigger an empathetic response reducing the chances of resolving the situation [398]. Interactions with other vitamins may be important either with deficits or excesses as we have implied for choline, and for example, the combination of low vitamin D intake with genetic predisposing defects, and, NAD supplies, and, altered relationships with microorganisms triggering autoimmune demyelination may be important in the pathogenesis of multiple sclerosis [399, 400]. Intracellular pellagra may also be caused by other mechanisms such as high NAD expenditure after physical, emotional, or chemical toxic stress or acute infection or inadvertent removal of key symbionts, such as after antibiotic use, or mutations that impair autophagosome functions such that at some times or for some people the dose may need to be higher than usually recommended. Stress from loss of relative status is a powerful driver of ill health and primates including man revert to a high glucose and low nicotinamide diet or addictive behaviours that as with stress also induce NNMT that will further lower nicotinamide levels [401, 402].

One should not, on the other hand, expose populations to “too much of a good thing” with excessive supplies of nicotinamide or choline or calories and thereby perturb NAD : NADH ratios in favour of NADH, creating a mirror image of pellagra, perhaps with an equally broad phenotype [403]. Modern disease patterns may be contemporary mirror images of the Neolithic meat transition when with less meat fertility rose but so did disease reducing longevity, whereas now as meat eating is on the increase longevity is increasing with fertility dropping and different chronic diseases emerging, and others such as TB rapidly decreasing.

Poor worldwide distribution of NAD(H) supplies makes little sense if oversupply is dangerous, particularly if undersupply is not only dangerous to those affected but leads to new symbionts or drug resistant microbes aided and abetted by the host in metabolic desperation that can then infect others who are more affluent and cause potentially epidemic pathology [403]. Our current behaviour that does not deal with this urgently “altruistically” may need the insight that as group sizes were enlarged we moved away from our hunter-gatherer roots of an egalitarian norm and a sharing of nicotinamide and methyl group rich meat. “Stinginess” may date from when the natural meat supply deteriorated and meat and the considerable wherewithal to produce it became treated as property. Fighting and migrating were preferred over starving, and trading may have promoted specialisation, competition, and inequality including of access to meat [404]. Resource allocation both of food and of medicines is culturally driven with a tension as to whether those that earn the most or those with the greatest need are given preference. The former is the usual modern default option but is a cultural “homo-economicus” evolutionary trap that needs to be consciously and socially corrected [405–407]. Fairer access to high quality energy sources allows all people the choice of moderation and a Hippocratic style diet and exercise regimen, and, access to parallel drug developments which have hormetic and preconditioning effects: that test the energy axis, induce autophagy and unfolded protein targeting and endoplasmic reticulum stress responses to recycle protein debris, thus preventing and “vaccinating” against disease [408–410]. New treatments that quickly supply energy, or save energy by inducing artificial hibernation, if employed after an unexpected insult such as trauma, and allow natural healing to take place, may also work best [411].

Humans have been exploring ways of preventing cancer and degeneration for decades whereas evolution has coped with episodes of NAD(H) deficiency and excess for several billion years. Now it may be time to learn lessons from the experts and find a Goldilocks energy-methylome-informatic-economy that matches supply and demand throughout life. Fewer energy tradeoffs or faulty signals may avoid lost robustness, shorter lives and loss of a fitness trait such as intellectual or physical capacity as seen with the degenerative AD and PD or the proliferative cancers, inflammatory and allergic diseases.

## Figures and Tables

**Table 1:** Three worlds that interact are largely human thermodynamic and cognitive niche constructions that are all heritable making us codirectors of our own and our collaborators evolution. There is a cost to this cumulative evolution that may include hypo- and hypervitaminosis, energy, and informatic states.

Thermodynamic and material	Personal	Information and society
Omnivorous diet	Conscious perception	Divisions of labour
Trading symbionts	Empathy	Trading
Shelter/clothes	Mental time travel	Information stores
	Know-how	
	Language	
	Nutrigenomics	
	Immunogenomics	
	Neuro/endocrine homeostasis	

Surplus energy for innovation of tools and technology and further harnessing of energy.
